# Individual Sweet Taste Perception Influences Salivary Characteristics After Orosensory Stimulation With Sucrose and Noncaloric Sweeteners

**DOI:** 10.3389/fnut.2022.831726

**Published:** 2022-05-25

**Authors:** Corinna M. Karl, Ana Vidakovic, Petra Pjevac, Bela Hausmann, Gerhard Schleining, Jakob P. Ley, David Berry, Joachim Hans, Martin Wendelin, Jürgen König, Veronika Somoza, Barbara Lieder

**Affiliations:** ^1^Christian Doppler Laboratory for Taste Research, Faculty of Chemistry, University of Vienna, Vienna, Austria; ^2^Department of Physiological Chemistry, Faculty of Chemistry, University of Vienna, Vienna, Austria; ^3^Joint Microbiome Facility of the Medical University of Vienna and the University of Vienna, Vienna, Austria; ^4^Department of Microbiology and Ecosystem Science, Division of Microbial Ecology, Centre for Microbiology and Environmental Systems Science, University of Vienna, Vienna, Austria; ^5^Department of Laboratory Medicine, Medical University of Vienna, Vienna, Austria; ^6^Institute of Food Science, University of Natural Resources and Life Sciences, Vienna, Austria; ^7^Symrise AG, Holzminden, Germany; ^8^Symrise Distribution GmbH, Vienna, Austria; ^9^Department of Nutritional Sciences, Faculty of Life Sciences, University of Vienna, Vienna, Austria; ^10^Leibniz Institute for Food Systems Biology at the Technical University of Munich, Freising, Germany; ^11^Chair of Nutritional Systems Biology, School of Life Sciences, Technical University of Munich, Freising, Germany

**Keywords:** sweet taste, saliva, mouthfeel, sucrose, rebaudioside M, neohesperidin dihydrochalcone, sucralose, oral microbiome

## Abstract

Emerging evidence points to a major role of salivary flow and viscoelastic properties in taste perception and mouthfeel. It has been proposed that sweet-tasting compounds influence salivary characteristics. However, whether perceived differences in the sensory properties of structurally diverse sweet-tasting compounds contribute to salivary flow and saliva viscoelasticity as part of mouthfeel and overall sweet taste perception remains to be clarified. In this study, we hypothesized that the sensory diversity of sweeteners would differentially change salivary characteristics in response to oral sweet taste stimulation. Therefore, we investigated salivary flow and saliva viscoelasticity from 21 healthy test subjects after orosensory stimulation with sucrose, rebaudioside M (RebM), sucralose, and neohesperidin dihydrochalcone (NHDC) in a crossover design and considered the basal level of selected influencing factors, including the basal oral microbiome. All test compounds enhanced the salivary flow rate by up to 1.51 ± 0.12 g/min for RebM compared to 1.10 ± 0.09 g/min for water within the 1st min after stimulation. The increase in flow rate was moderately correlated with the individually perceived sweet taste (*r* = 0.3, *p* < 0.01) but did not differ between the test compounds. The complex viscosity of saliva was not affected by the test compounds, but the analysis of covariance showed that it was associated (*p* < 0.05) with mucin 5B (Muc5B) concentration. The oral microbiome was of typical composition and diversity but was strongly individual-dependent (permutational analysis of variance (PERMANOVA): *R*^2^ = 0.76, *p* < 0.001) and was not associated with changes in salivary characteristics. In conclusion, this study indicates an impact of individual sweet taste impressions on the flow rate without measurable changes in the complex viscosity of saliva, which may contribute to the overall taste perception and mouthfeel of sweet-tasting compounds.

## Introduction

The flavor perception of sweeteners involves not only gustation and olfaction but also the overall tactile impression called mouthfeel. The origin of the multidimensional sensation of mouthfeel has not yet been fully characterized, but several contributing factors have been identified. In addition to the organoleptic, textural, and surface properties of foods or beverages [reviewed by Guinard and Mazzucchelli (1)], oral physiology, especially saliva with its lubricating and transporting properties, plays a major role in the overall flavor, mouthfeel, and the so-called afterfeel (2, 3). In particular, the flow rate and rheological properties of saliva have been reported to be related to the difference in the flavor impression of a compound (4). Salivary flow, which may act against oral surface irrigation (5), can be stimulated mechanically by chewing and through various taste stimuli depending on the concentration of the stimulus. Although the most potent activation of salivary flow has been shown with sour-tasting citric acid (6–8), sweet taste has also been associated with the stimulation of salivary flow (9, 10), but the interaction of salivary characteristics, including flow rate, and sweet taste perception remains to be elucidated. In addition, a complex interplay of mechanisms affects salivary flow, depending, for example, on the nature of the molecules, food matrices, or interactions with salivary compounds, making it difficult to generalize the effect of taste stimuli on salivary flow (11).

The lubricating effects of saliva are determined not only by the flow rate but also by the viscous and elastic components; however, the effects of different stimuli on the viscoelastic properties of saliva have not yet been completely clarified. A previous study by Stokes and Davies (12) compared the viscoelasticity after stimulation with citric acid, water, and chewing gum. They showed that the viscoelasticity of whole oral saliva was associated with the type of stimulus independent of the induced flow rate, while the viscosity of saliva after different stimuli was similar (12). Later, Davies et al. (4) compared the rheology of saliva after stimulation with ice tea, fizzy cola, sparkling water, chewing gum, mint, or water. The results showed that ice tea and cola induced the highest salivary flow rate and higher elasticity compared to chewing gum or water but had similar overall viscosity. The authors concluded that the elasticity of saliva is independent of the flow rate and that the rheology of saliva can affect the sensory properties, including mouthfeel, of beverages (4). Other compositional factors that determine the rheological properties and flow of saliva are the enzymes α-amylase (8, 11, 13, 14) and cystatin S. The latter one is mainly secreted from submandibular and sublingual glands (15, 16). Also, mucin 5B (Muc5B), a major mucin protein in saliva (17), and the pH of saliva, which normally ranges from 6.7 to 7.4 (18), influence the viscoelasticity of saliva. If saliva has higher viscosity and lower elasticity, it cannot form an optimal salivary pellicle (19). The formation of a salivary pellicle is important for lubrication and protection of the oral surface and involves salivary proteins. Thus, the interaction of sweet compounds with salivary proteins may influence chemosensation (3). The mucin Muc5B is an important protein involved in the formation of a salivary pellicle and has been associated with taste perception and astringency (20, 21). Recently, the aggregation of mucosal pellicles by polyhydroxyphenols (tannins), leading to the dissociation of the protein Muc1, has been proposed as the underlying mechanism to sense astringency (22). A similar disruption of the mucosal pellicle by polyphenolic sweeteners such as neohesperidin dihydrochalcone (NHDC) is conceivable but has not been proven yet. While mechanical stimulation leads to reduced elasticity of the saliva, stimulation with citric acid leads to secretion of more elastic saliva (12), leading to the conclusion that different types of stimuli induce secretion from distinct types of glands, which affect the elasticity of saliva (12, 23). In the case of sucrose, enhanced concentration was shown to increase the viscosity rating of aqueous solutions (24). At this point, it is not clear whether this perceived viscosity is based mainly on cross-modal effects in the brain that associate increased sweet taste with higher viscosity or on actual changes in perceived mouthfeel.

In addition, the influence of the oral microbiota on the interplay of mouthfeel, taste perception, and salivary parameters has been proposed (25, 26). For example, the ecological effect of the oral microbiota not only depends on sugar intake but also is influenced by the taste phenotype of the host through allelic variation in the *TAS1R1* and *GNAT3* genes or by the salivary flow rate (25). Previous studies also provided evidence that the oral microbiota is associated with the PROP status of individuals, reflecting their ability and sensibility to taste bitter 6-*n*-propylthiouracil based on their genetic variation in *TAS2R38* (26, 27). However, to date, only limited data exist on the association between taste perception and the oral microbiota.

In summary, emerging evidence suggests that salivary flow and viscoelastic properties play a major role in taste perception and mouthfeel. However, it is not clear whether and how differences in the sensory properties of structurally diverse sweet-tasting compounds contribute to salivary flow and saliva viscoelasticity as part of mouthfeel and overall taste perception. In addition, the role of the oral microbiome in the interplay of saliva, taste, and mouthfeel remains largely unknown. Thus, we hypothesized in this study that the structurally and sensorially diverse sweet tasting compounds sucrose and the noncaloric rebaudioside M (RebM), sucralose, and NHDC compounds differentially affect salivary flow and the complex viscosity of saliva. Several factors that might influence salivary characteristics were considered, namely, body mass index (BMI), age and sweet threshold of test subjects, individual sweet taste perception of the test compounds, pH, α-amylase activity, cystatin S, Muc5B, storage modulus (*G*’), and phase angle (δ) of basal saliva. Moreover, we investigated whether there was a relationship between the composition of the basal oral microbiota, salivary properties, and sweet taste and overall flavor perception.

## Materials and Methods

### Test Compounds and Test Subjects

**FIGURE 1:**
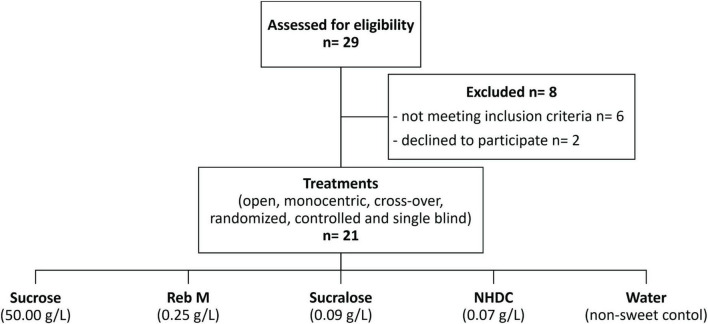
Flow diagram of the study population and the sucrose, rebaudioside M (RebM), sucralose, and neohesperidin dihydrochalcone (NHDC) treatments at concentrations equivalent to 5% (w/v) sucrose, and water as non-sweet volume control. All of them were tested in a randomized, single blind crossover design.

Four structurally and sensorially diverse sweet-tasting compounds, namely, NHDC (>96%, FG; Sigma-Aldrich, Steinheim, Germany), RebM (90%; Symrise AG, Holzminden, Germany), sucralose (>98%; Symris AG, Holzminden, Germany), and sucrose (AGRANA Zucker GmbH, Vienna, Austria) were selected as test compounds (refer to Table 1 for the corresponding IUPAC nomenclature and structures). The compounds and their concentrations were selected based on a previous sensory study by Karl et al. (28), in which sweet taste affecting compounds were sorted into three main clusters based on their sensory properties (28). For the present study, a representative compound was selected from each cluster in addition to the sweet reference compound, sucrose. The concentration of the compounds was chosen to be equivalent to the sweet taste of 5% (w/v) sucrose, with 0.07 g/L NHDC, 0.25 g/L RebM, 0.09 g/L sucralose, and 50.0 g/L sucrose according to Karl et al. (28) (see also Figure 1). All compounds were dissolved in Viennese tap water (pH = 7.88 ± 0.02) and thus an equivalent volume of water was applied as a taste-neutral volume control. Viennese tap water was chosen because the local test subjects are accustomed to its taste and it provides a stable quality.

**TABLE 1:** IUPAC names and chemical structures of the test compounds.

Test compounds	IUPAC computed by Lexichem TK 2.7.0 (PubChem release 2021.05.07)	Structure
Sucrose	(2R,3R,4S,5S,6R) -2-[(2S,3S,4S,5R) -3,4-dihydroxy -2,5-bis(hydroxymethyl)oxolan -2-yl]oxy-6-(hydroxymethyl)oxane-3,4,5-triol	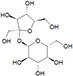
Reb M	[(2S,3R,4S,5R,6R) -5-hydroxy -6-(hydroxymethyl) -3,4-bis [[(2S,3R,4S,5S,6R) -3,4,5-trihydroxy -6-(hydroxymethyl)oxan -2-yl]oxy]oxan-2-yl] (1R,4S,5R,9S,10R,13S) -13-[(2S,3R,4S,5R,6R) -5-hydroxy -6-(hydroxymethyl) -3,4-bis [[(2S,3R,4S,5S,6R) -3,4,5-trihydroxy -6-(hydroxymethyl)oxan -2-yl]oxy]oxan-2-yl]oxy -5,9-dimethyl -14-methylidenetetracyclo [11.2.1.01,10.04,9] hexadecane -5-carboxylate	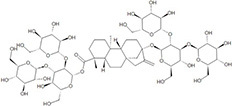
Sucralose	(2R,3R,4R,5R,6R) -2-[(2R,3S,4S,5S) -2,5-bis(chloromethyl) -3,4-dihydroxyoxolan-2-yl] oxy -5-chloro -6-(hydroxymethyl)oxane -3,4-diol	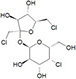
NHDC	1-[4-[(2S,3R,4S,5S,6R)-4,5-dihydroxy-6-(hydroxymethyl)-3-[(2S,3R,4R,5R,6S) -3,4,5-trihydroxy -6-methyloxan -2-yl] oxyoxan -2-yl] oxy-2,6-dihydroxyphenyl] -3-(3-hydroxy -4-methoxyphenyl) propan-1-one	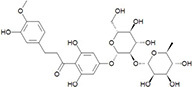

The number of test subjects was estimated using a power analysis with the software G*Power 3.1 based on the study by Neyraud et al. (6). The study showed an increase in salivary flow after stimulation with sweet-tasting carbohydrates, from which an effect size of 0.94 was calculated, leading to the total number of at least 17 subjects with α = 0.05 and 95% power.

The ethics committee of the University of Vienna (reference no. 00421; 2019) approved this study, and all test subjects gave written informed consent. The test subjects were recruited from the University of Vienna and the surrounding area. In total, 29 test subjects participated in the screening. Participants had to be between 18 and 60 years of age and in good general health. The exclusion criteria included smoking, pregnancy, or breastfeeding, chronic conditions with teeth or gingivitis, permanent medication, antibiotics treatment within the last 2 months, diagnosed anosmia or ageusia, viral or bacterial infections within the last 3 weeks, alcohol or drug addiction, known allergies to any of the test substances, and a basal salivary flow rate of less than 0.3 g/min. Age, body weight [Soehnle Industrial Solutions GmbH, Backnang, Germany (61,350), max: 150 kg; accuracy: 0.1 kg], and body height [stadiometer from Seca, Hamburg, Germany, max: 2.10 m, accuracy: 0.01 m] were recorded, and individual BMI [BMI = weight [kg]/height^2^ [m^2^]] was calculated. Of the 29 test subjects enrolled, six were excluded after screening for not meeting the inclusion criteria and two withdrew their consent to participate. A total of 21 test subjects completed all five treatments (see also Figure 1).

### Study Design

**FIGURE 2:**
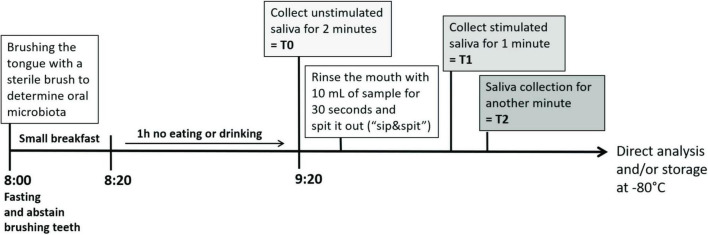
Study design—timeline and overview of a study day with sampling of oral microbiome, unstimulated (T0) and stimulated saliva in the first (T1) and second (T2) min after stimulation with one of the test compounds. The procedure was repeated for each test compound on a separate day with at least 3 days apart.

The study design was an open, single-centered, randomized, crossover, single blinded, and controlled study. Each test substance was tested on a separate study day and by each test subject (see Figure 1) to prevent carryover effects of the test substances, at least 3 days apart. Test subjects completed all test days within 3–6 weeks between May and October 2019. On each study day, saliva samples were collected at 9 a.m. at three time points (Figure 2) in 5 ml tubes (Carl Roth, Karlsruhe, Germany). Because salivation and salivary parameters can be easily affected, for example by time of day, stimulation, and diet (29, 30), the sampling procedure was standardized and training of saliva collection during screening was performed. Test subjects with less than 0.3 g/min of unstimulated saliva were excluded from the study as this volume is described as the threshold of the normal range of salivation (31). A flow rate of 0.7 g/min of stimulated saliva was required to determine all parameters. On each study day, test subjects were asked to arrive in a fasting state and without brushing their teeth at 8 a.m. at the research facility. First, approximately 1 cm^2^ of one side of the tongue dorsum was brushed with a sterile swab (ESwab 480C, Copan Diagnostics, Inc., Murrieta, CA, United States) according to the manual of procedures for the human microbiome project, version 12.0^1^ to determine the basal composition of the oral microbiome in the area of the fungiform papillae to see if the individual oral microbiota is constant over the study time and might influence taste perception or mouthfeel attributes. Samples were frozen at −80°C until analysis. After swabbing, the test subjects consumed the standardized breakfast provided (one pretzel with 10 g of butter and up to 300 ml of water), followed by brushing the teeth with a flavor-neutral toothpaste composed solely of calcium carbonate (obtained from a local pharmacy) and tap water. Test subjects had to abstain from eating and drinking for 1 h before starting saliva collection. Unstimulated resting saliva was collected for 2 min (T0). After the collection of unstimulated saliva, test subjects rinsed their mouth with 10 ml of the sample for 30 s and spat out the entire sample. Stimulated saliva was then collected after spitting out the sample separately for the first min (T1) and the second min (T2). All saliva samples were kept on ice immediately after collection. Aliquots of saliva samples were frozen at −80°C for subsequent analysis of protein content, α-amylase activity, cystatin S, and Muc5B. The flow rate, pH, and viscoelastic parameters of saliva were analyzed directly after collection.

### Sensory Evaluation

The screening included the determination of the individual sweet taste threshold and the sweet intensity rating of the test compounds in a fully equipped sensory laboratory. The sweet threshold level of the test subjects was determined with increasing sucrose concentrations from 0.34 to 12.00 g/L according to DIN EN ISO 3972:2013-12 (32) described in detail by Höhl and Busch-Stockfisch (33). Moreover, the sweet taste intensity for each test compound was rated on an unstructured continuous scale [0–10] after pre-tasting five sweet solutions with 0, 6, 20, 50, and 100 g/L of sucrose as “not at all” to “very intensive” sweet taste.

### Determination of Salivary Parameters

#### Salivary Flow

Salivary flow was determined gravimetrically on an analytical scale (Satorius AG, Göttingen, Germany 224i-1S, with a reproducibility of 0.1 mg) immediately after sample collection. For this purpose, the test subjects were asked to salivate in individually pre-weighed 5 ml tubes for exactly 2 (T0) or 1 min (T1 and T2). Data are presented as salivary flow in g/min.

#### pH

Salivary pH was measured using 20 μl of fresh saliva samples with a pocket pH meter (PH60F, Apera Instruments GmbH, Wuppertal, Germany; pH ± 0.01, measuring range pH -2.0–16.0).

#### Total Protein Content

Total protein content in saliva was measured according to Bradford (34). Samples were centrifuged at 3 000 × *g* for 15 min at 4°C, and the supernatant was mixed with an equal volume of RIPA lysis buffer (50 mM tris(hydroxymethyl)-aminomethane, 25 mM sodium chloride, 1 mM ethylenediaminetetraacetic acid, 1 mM sodium fluoride). Samples, or 0.025–1.0 mg/ml bovine serum albumin (Thermo Scientific, Rockford, United States) as standards, were mixed with Bradford color reagent (2.5 mg Coomassie Blue G-250 with 150 ml methanol and 50 ml phosphoric acid, filled with ddH_2_O to 1 L) (1:100) and incubated for 15 min. The absorbance of each sample or standard was measured in triplicate using a multimode plate reader (Tecan Infinite M200, Tecan Group Ltd., Männedorf, Switzerland) at 595 nm and a reference wavelength of 850 nm and the protein concentration was presented as mg/ml.

#### α-Amylase Activity

The α-amylase activity in saliva was determined using an enzymatic hydrolysis assay (35, 36) with three technical replicates for each saliva sample. The saliva samples were centrifuged at 3,000 × *g* for 15 min at 4°C, and the supernatant was used for further analysis. An equal volume of 1% (w/v) starch solution was added to the saliva samples and, after exactly 3 min, one volume of color reagent solution was added. The color reagent solution consisted of 1.0 g of 3, 5-dinitrosalicylic acid with 30.0 g of sodium potassium tartrate tetrahydrate and 20 ml of 2 M NaOH solution in 100 ml water. The reaction was stopped by adding five volumes of ddH_2_O. The α-amylase activity was determined by the detection of maltose cleaved from the starch. Maltose reduces 3, 5-dinitrosalicylic acid to 3-amino-5-nitrosalicylic acid, causing a shift in the absorbance at 540 nm, which was analyzed using a multimode plate reader (Tecan Infinite M200, Tecan Group Ltd., Männedorf, Switzerland). Quantification of enzymatic starch cleavage was accomplished using an external standard curve (maltose 0.125–5.0 μmol/ml), and the amount of maltose produced in the presence of salivary α-amylase was determined by extrapolation after subtraction of the blank (ddH_2_0 instead of maltose solution) from the standard and sample values. The results were normalized to total protein content and presented as μmol of maltose per mg protein released per minute (μmol/mg protein/min).

#### Cystatin S

The amount of cystatin S in saliva was determined using a quantitative colorimetric sandwich-ELISA kit (abx 151234, Abbexa Ltd, Cambridge, United Kingdom, range: 0.156–10 ng/ml, sensitivity: <0.066 ng/ml). The saliva samples were centrifuged at 3 000 × g for 15 min at 4°C, and the supernatant was analyzed in duplicates according to the manufacturer’s protocol. Absorbance values were detected at 450 and 650 nm as the reference wavelength using a multimode plate reader (Tecan Infinite M200, Tecan Group Ltd., Männedorf, Switzerland). The results were normalized to the amount of protein and were presented as μg per mg protein (μg/mg protein).

#### Rheological Properties

Rheological properties, such as complex viscosity (η), storage modulus (*G*′) representing the elastic part, loss modulus (*G*″) representing the viscous part, and the phase angle (δ) representing the relative saliva viscoelasticity, were measured with a regularly calibrated oscillating rheometer (Kinexus, Malvern Panalytical GmbH, Kassel, Germany). A 20-mm diameter plate–plate probe (plate PU-20) was used with a gap of 0.5 mm. The saliva samples were kept on ice and measured directly after collection. Measurements were carried out using a frequency sweep at 36°C in the linear region of an amplitude sweep at a strain of 0.5% and a frequency range from 0.6 to 0.1 with six linear measuring points. SDS was not added to the samples as it is known that this can cause unfolding and dissociation of salivary proteins, affecting the aggregation state of mucins (37) and thereby influencing the structure and viscosity of saliva. Prior to performing the experiments, the method was established and data were validated using an amplitude sweep at a frequency of 0.5 with linear moduli up to 1% (see Supplementary Table 1). A strain of 0.5% was chosen as the software determined that this was the optimum strain for the selected frequency sweep, and the frequency range was chosen according to the low-interference area of the device. As the measurement time was less than 3 min, it was not necessary to cover the samples to avoid evaporation. The mean of two to three measurements of each salivary sample [*n* = 21 for each test compound (see Figure 1) and the three time points of saliva collection (see Figure 2)] was used as the value for each of the rheological parameters. The results are presented as Pa s for complex viscosity η, Pa for storage modulus *G*′, Pa for loss modulus *G*″, and ° for phase angle δ.

#### Determination of Muc5B

A quantitative colorimetric sandwich-ELISA kit (abx 250243, Abbexa Ltd., Cambrige, United Kingdom, range: 0.625–40 ng/ml, sensitivity: 0.38 ng/ml) was used to determine the amount of the glycoprotein Muc5B in saliva. After centrifuging saliva samples at 3,000 × g for 15 min at 4°C to remove debris, the supernatant was analyzed in duplicate according to the manufacturer’s manual. Absorbance values were detected at 450 and at 650 nm as the reference wavelength using a multimode plate reader (Tecan Infinite M200, Tecan Group Ltd., Männedorf, Switzerland). The results were normalized to the amount of protein and presented as ng per mg protein (ng/mg protein).

#### Oral Microbiome Composition

The oral microbiome was analyzed by the 16S rRNA gene amplicon sequencing analysis performed at the Joint Microbiome Facility (project ID JMF-1908-4) using a previously described two-step PCR approach (38). Briefly, DNA from tongue swabs and control swabs was extracted using the QIAamp DNA Microbiome kit (Qiagen) following the manufacturer’s instructions. In the first PCR step, the V4 region of bacterial and archaeal 16S rRNA genes was amplified (35 cycles) with the 515F and 806R primers (39, 40), which were modified with linker sequences [UDB-H12 barcoding approach (38)]. In the second step, the amplicons were barcoded (eight cycles) in a unique dual (UDD-H12) setup. After the first step PCR and after barcoding, the samples were purified and normalized over the SequalPrep™ Normalization Plate kit (Invitrogen) using the Biomek^®^ NXP Span-8 pipetting robot (Beckman Coulter). Barcoded samples were pooled and concentrated on columns (Analytik Jena), and the indexed sequencing libraries were prepared from these amplicon pools with the Illumina TruSeq Nano kit, as described in a previous study (38). Amplicon pools were sequenced in a paired-end mode (2 × 300 nt; v3 chemistry) on an Illumina MiSeq following the manufacturer’s instructions. The workflow systematically included four negative controls (PCR blanks, i.e., PCR-grade water as template) for all 90 samples sequenced. Amplicon pools were extracted from the raw sequencing data using the FASTQ workflow in BaseSpace (Illumina) with default parameters. Further, demultiplexing was performed with the python package demultiplex (Laros JFJ^2^), allowing one mismatch for barcodes and two mismatches for linkers and primers each. Amplicon sequence variants (ASVs) were inferred using the DADA2 R package [3] applying the recommended workflow (41). The resulting FASTQ reads were trimmed at 145 nt with the allowed expected error of 2. ASVs were classified using SINA version 1.2.11 (42) and the SILVA database SSU Ref NR 99 release 132 (43) using default parameters. All generated amplicon sequencing data were deposited to the Sequence Read Archive (SRA) and can be found under the BioProject ID PRJNA726851.

### Statistical Analysis

Data calculation and statistical analysis of the salivary characteristics were performed using MS Excel 16.0, GraphPad Prism 8.0, and IBM SPSS Statistics 26. All data sets were tested for normality with the Shapiro–Wilk test. To assess compound- and time-dependent effects, a repeated measures two-way analysis of variance (ANOVA) followed by Tukey’s *post-hoc* test for dependent data was performed and checked for normally distributed residuals. To evaluate differences between the treatments, the data were normalized to the respective baseline value at T0 (ΔT1, ΔT2) and to the volume control water [ΔΔTx = (Tx–T0)–(Tx−T0)_H2O_]. To evaluate the impact of the test compounds and selected influencing factors on the salivary flow and the complex viscosity (N* complex), a repeated measures analysis of covariance (ANCOVA) was carried out using SPSS. The ANCOVA was used to see if there were effects of selected and potentially influencing metric covariates on salivary flow and complex viscosity either dependent (intrasubject factor) or independent (intermediate subject effects) of the test compounds. Several factors that may influence salivary characteristics were included as covariates, namely, the BMI, age, and sweet threshold of the test subjects, individual sweet perception of the test compounds, pH, α-amylase activity, cystatin S, Muc5B, storage modulus (*G*′), and phase angle (δ) of basal saliva. The Pearson’s product moment correlation was applied for a correlation analysis. To compare differences in the perceived sweet taste of the test compounds, a one-way ANOVA with Tukey’s *post-hoc* test was performed in GraphPad Prism. Unless otherwise indicated, data are presented as mean ± standard error of the mean (SEM). Differences were considered as significant at *p* < 0.05 and with *p* < 0.1 as a trend. In all figures and tables, significant differences were marked with either asterisk or different letters.

To test for associations between various recorded descriptive and physiological parameters and the tongue dorsum microbiome composition, the ASV table was rarefied to the minimum sample depth (3,650 sequences) using the “rrarefy” function from the R package vegan^3^ (44). PERMANOVA was performed with the “adonis” function (45) of the R package vegan. Bray–Curtis dissimilarity was used as a dissimilarity metric. Otherwise, default parameters were used.

## Results

### Salivary Flow

The characteristics of the test subjects, including the sweet taste threshold [g/L sucrose], are summarized in Table 2. The distribution of the sweet sensitivity threshold is shown in Supplementary Figure 1.

**TABLE 2:** Characteristics of the study participants.

Test subjects	Total *n* = 21	
	Mean	±SD
Age [y]	26.57	±5.07
Female/Male	10/11	
Weight [kg]	71.89	±11.84
Height [m]	1.77	±0.09
BMI [kg/m^2^]	22.74	±2.18
Threshold sweet taste [g/L sucrose]	2.48	±1.54

**FIGURE 3:**
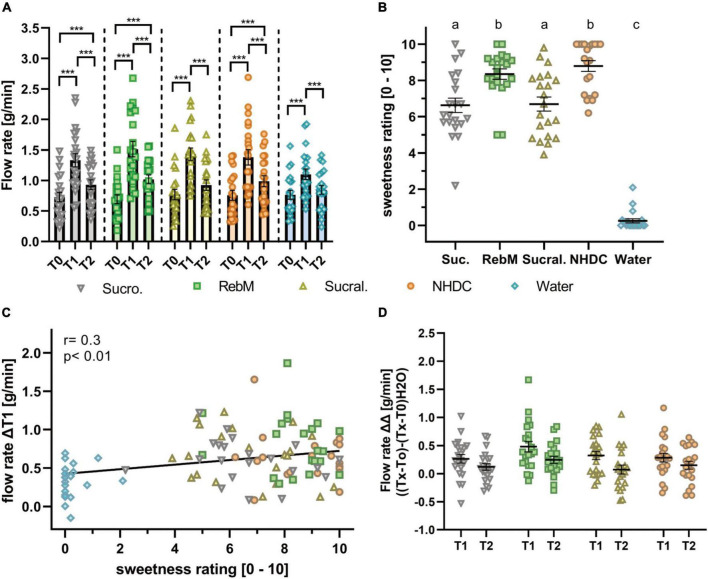
**(A)** Flow rate (g/min) of unstimulated (T0) and stimulated saliva in the 1st (T1) and 2nd (T2) min after stimulation with the test solutions sucrose (suc.), RebM, sucralose (sucral.), NHDC, and water as control; presented as mean ± standard error of the mean (SEM); tested for difference with a two-way ANOVA and Tukey’s *post-hoc* test; significant differences between measurement time points (T0-1-2) are marked with ***(*p* < 0.001). **(B)** Individually, perceived sweet taste rating (0-10) of the test solutions. Significant differences were tested with a one-way ANOVA with Tukey’s *post-hoc* test and are labeled with different letters. **(C)** Pearson product moment correlation of sweet taste rating and ΔT1 flow rate (g/min). **(D)** Normalized flow rate, calculated as [g/min] [(Tx-T0)-(Tx-T0)_H2O_] after stimulation with the test solutions, presented as mean ± SEM. All figures include single values (*n* = 21) for each test compound.

First, the impact of oral stimulation with the selection of sensorially and structurally different sweet-tasting compounds adjusted for sweet taste, namely, sucrose, RebM, sucralose, and NHDC, on salivary flow with water as taste-neutral volume control was investigated. The basal unstimulated salivary flow rate (T0) between the test days did not differ significantly (*p* > 0.05). All tested stimuli, including water, enhanced the salivary flow during the 1st min after stimulation (T1): sucrose 1.33 ± 0.11 g/min, RebM 1.51 ± 0.12 g/min, sucralose 1.43 ± 0.10 g/min, NHDC 1.38 ± 0.12 g/min, and water 1.10 ± 0.09 g/min (Figure 3A). In comparison to water, salivary flow was significantly enhanced after stimulation with RebM and sucralose at T1. In the 2nd min after stimulation (T2), salivary flow was decreased compared to T1 after stimulation with each treatment (T2: sucrose 0.93 ± 0.08 g/min, RebM 1.02 ± 0.07 g/min, sucralose 0.92 ± 0.09 g/min, NHDC 0.99 ± 0.09 g/min, and water 0.84 ± 0.07 g/L) but was still increased in comparison to the basal flow rate except for sucralose and the water control (Figure 3A). There was no difference in salivary flow stimulation at T2 between the compounds.

The concentration of the test compounds was selected to reach the sweet taste level equivalent to 5% sucrose, based on the rating of trained panelists, according to Karl et al. (28). However, the sensorially naïve test subjects in this study rated NHDC and RebM to be sweeter than sucralose and sucrose (Figure 3B). As individually perceived sweet taste between the compounds differed, a more detailed look was taken at the relationship between individually perceived sweet taste and the salivary flow rate. As expected, sucrose, RebM, sucralose, and NHDC were rated to be significantly sweeter than the volume control water. In addition, there was a moderate positive correlation between individually perceived sweet taste and the salivary flow rate within the 1st min (*r* = 0.3, *p* < 0.01, Figure 3C).

To investigate the hypothesis that sensorially and structurally distinct sweet-tasting compounds will lead to differences in the flow rate, the stimulated salivary flow was normalized to the unstimulated flow rate on each test day and to the effect of the volume control water (ΔΔT_x_) (see Figure 3D). Using a repeated measures ANCOVA, which passed the Mauchly test for sphericity (*p* > 0.05), the effect of the test compounds and several potentially influencing factors as covariates, namely, the basal values (T0) of pH, α-amylase activity, cystatin S content, the sweet threshold, body height, BMI, and age of test subjects on the salivary flow rate (ΔΔT1) was evaluated. The sweet taste evaluation was excluded as we determined differences in the mean sweet taste evaluation of the test compounds by our untrained panelists. None of the covariates influenced the salivary flow rate in dependence of the test compounds (intrasubject factor, see Supplementary Table 2). However, independent of the treatment (intermediate subject effects, see Supplementary Table 2), the body height and the interaction of α-amylase activity with the sweet taste threshold showed a trend (*p* < 0.1) to affect the flow rate. Table 3 shows the unadjusted and covariate-adjusted means of the salivary flow rate (ΔΔT1). No significant difference was found between the test substances for unadjusted or adjusted salivary flow values.

**TABLE 3:** Unadjusted values and covariate-adjusted means (±SD/SE) of the normalized salivary flow rate ΔΔT1 [g/min] = [(Tx T0)-(Tx-T0)_H2O_] after stimulation with each test solution analyzed by means of an repeated measures ANCOVA with the basal level of α-amylase activity, cystatin S, pH, threshold, body height, body mass index (BMI), and age of participants as covariates.

ΔΔT1 flow rate [g/min]		Unadjusted		Adjusted	
	*N*	Mean	±SD	Mean	±SE
Sucrose	21	0.26	0.35	0.32	0.06
RebM	21	0.48	0.43	0.53	0.11
Sucralose	21	0.32	0.33	0.37	0.08
NHDC	21	0.28	0.36	0.32	0.11
*p*-value		0.532		0.215	

### Viscoelastic Properties of Saliva

**FIGURE 4:**
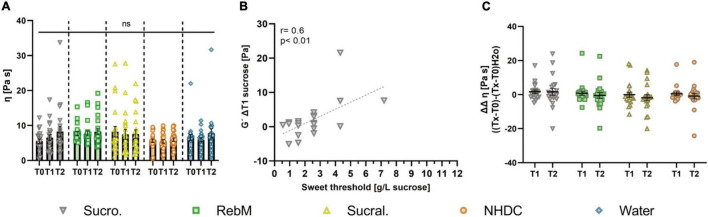
**(A)** Complex viscosity η (Pa s) of unstimulated (T0) and stimulated saliva in the 1st (T1) and 2nd (T2) min after stimulation with test solutions sucrose (suc.), RebM, sucralose (sucral.), NHDC, and water as control; presented as mean ± SEM; tested for difference with two-way ANOVA and Tukey’s *post-hoc* test; (ns) no significant results were detected. **(B)** Normalized complex viscosity η, calculated as (Pa s) [(Tx-T0)-(Tx-T0)_H2O_] of saliva samples after stimulation with each test solution; presented as mean ± SEM, *n* = 21. **(C)** Pearson product moment correlation for storage modulus *G*′ (Pa) ΔT1 after stimulation with sucrose and the sweet taste threshold (g/L sucrose).

Next, we investigated the impact of oral stimulation with sucrose, RebM, sucralose, and NHDC on the viscoelastic properties of saliva. A representative measurement of *G*′, *G*″, and phase angle (δ) against the frequency of one saliva sample with two repetitions is shown in Supplementary Figure 2. Furthermore, Figure 4A shows the mean complex viscosity (η) of saliva before (T0) and at the 1st (T1) and 2nd (T2) min after stimulation with the test solutions. The complex viscosity of basal, unstimulated saliva samples did not differ throughout the different study days. In contrast to our hypothesis, oral stimulation with none of the test compounds resulted in differences in η (Figure 4A). Also, no differences were found for the storage and loss modulus (*G*′ and *G*″) of unstimulated and stimulated saliva (see Supplementary Table 3 for raw data). The sweet taste threshold of the test subjects correlated with *G*′ (ΔT1) of saliva after stimulation with sucrose (*r* = 0.6, *p* < 0.01, Figure 4B).

The consideration of potentially influencing covariates was evaluated using a repeated measures ANCOVA for the values of η normalized to the water control (ΔΔT1) (see Figure 4C). The ANCOVA included as covariates the basal values (T0) of Muc5B, pH, α-amylase, storage modulus (*G*′) and phase angle (δ) of saliva as well as sweet threshold and age of test subjects and passed the Mauchly test for sphericity (*p* > 0.05). The results (Supplementary Table 4) of the intrasubject factor showed that, depending on the test compounds, the interaction of *G*′ with the sweet threshold influenced complex viscosity η of the saliva (*p* < 0.05). The intermediate subject effects showed that, independent of the test compound, the basal amount of Muc5B significantly influenced the complex viscosity (*p* < 0.05). There was no effect of the pH of saliva samples or age of test subjects on complex viscosity (*p* > 0.1). Overall, no significant differences between the test compounds were found for either the unadjusted or the covariate-adjusted means of the complex viscosity (Table 4).

**TABLE 4:** Unadjusted values and covariate-adjusted means (±SD/SE) of normalized complex viscosity η values (ΔΔT1 [Pa s] = [(Tx T0)-(Tx-T0)_H2O_]) of saliva samples after stimulation with each test solution analyzed by means of an repeated measures ANCOVA with the basal level of mucin 5B (Muc5B), storage modulus *G*′, phase angle δ, pH, α-amylase activity, threshold, and age of participants as covariates.

ΔΔT1 η [Pa s]		Unadjusted		Adjusted	
	*N*	Mean	±SD	Mean	±SE
Sucrose	21	1.65	4.70	0.72	1.13
RebM	21	0.75	5.92	–0.41	1.56
Sucralose	21	–0.08	6.99	–0.17	1.35
NHDC	21	0.44	4.38	–0.44	1.25
*p*-value		0.699		0.565	

### Oral Microbiome

**FIGURE 5:**
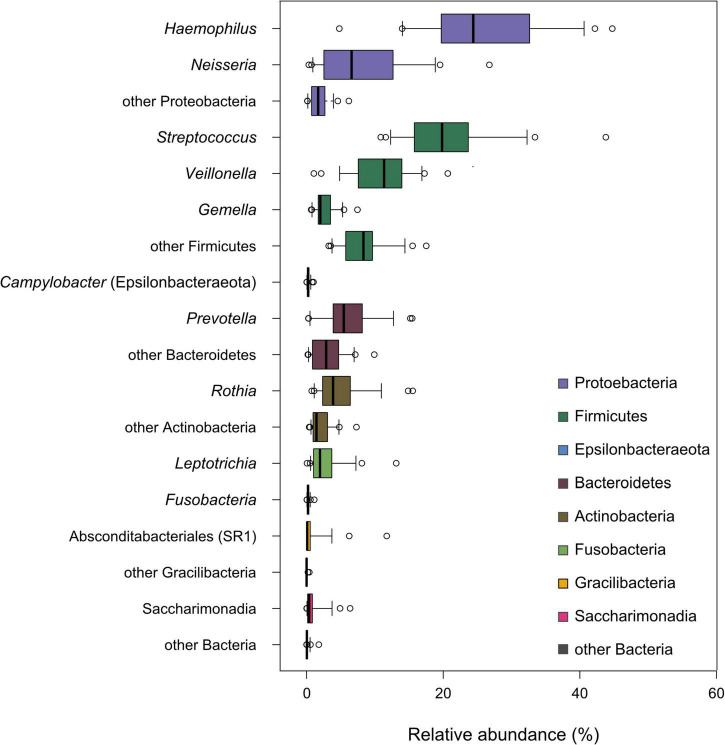
Tongue dorsum microbiome composition across all study participants. The cumulative relative abundance per individual of amplicon sequence variants (ASVs) classified as select, dominant bacterial genera is shown in the box plots. The phylum-level taxonomic classification of these genera is indicated by the box color.

Finally, we addressed the question of whether the individual basal oral microbiome is associated with the sweet taste perception and the analyzed salivary characteristics of unstimulated and stimulated saliva as well as their related parameters, including age, BMI, and sweet taste threshold. Tongue dorsum microbiome composition was neither influenced by the sampling day and tongue side nor by age, sex, and BMI of the test subjects, the sweet recognition thresholds, or basal salivary parameters (PERMANOVA, *p* > 0.05), except for the pH of unstimulated saliva (T0), which displayed a weak but statistically significant correlation with the observed microbiome composition (*R*^2^ = 0.03, *p* < 0.01; data not shown). All analyzed samples, regardless of the sampled individuals and the sample collection time point, displayed a composition and a diversity typical of human oral microbiome samples previously obtained from tongues of healthy individuals (46–48). The microbiomes of tongue samples from the 21 subjects investigated here were colonized by bacteria phylogenetically affiliated with eight different phyla, with *Proteobacteria* being the most abundant, followed by *Firmicutes*, *Bacteroidetes*, *Actinobacteria*, *Fusobacteria*, *Saccharimonadia*, *Gracilibacteria*, and *Epsilonbacteraeota*. Specifically, samples were dominated by ASVs affiliated with the genera *Haemophilus, Neisseria, Streptococcus, Veillonella, Gemella, Prevotella, Rothia*, and *Leptotrichia* (see Figure 5). Oral microbiota composition was strongly individual-dependent (PERMANOVA: *R*^2^ = 0.76, *p* < 0.001) and stable over the testing period, which lasted at least 14 days, by comparing the samples obtained on test days 1 and 5 of each test subject (see Supplementary Figure 3). All analyses of the association between microbiome composition and physiological parameters determined in saliva samples of the test subjects were constrained (strata) to the test subjects.

## Discussion

In this study, we investigated the influence of the stimulation with three structurally diverse sweeteners, namely, sucralose, RebM, and NHDC, compared to the most abundant sweet compound sucrose, on salivary flow and the viscoelastic properties of saliva in 21 healthy, adult test persons. Furthermore, we investigated whether there is an association between the individual oral microbiome and the salivary characteristics or sweet taste perception.

Our results show that the stimulation with all test compounds, as well as the water control, enhanced saliva flow compared to unstimulated saliva, with RebM leading to the most long-lasting effect. However, we not only focused on salivary flow itself, but also considered several influencing factors as covariates. Thus, by applying a repeated measures ANCOVA, we excluded the impact of several potentially influencing factors, namely, the basal cystatin S content, pH of saliva, and BMI and age of the test subjects. Those factors were selected as previous studies showed associations to salivary flow and overall taste perception (15, 49–52) although complex relationships with several factors have not been addressed so far. Our results show that, independent of the test compounds, salivation tended to be associated with body size and basal α-amylase activity. In addition, individually perceived sweet taste of the compounds showed a moderate but significant correlation to the salivary flow rate, which supports the fact that sweet stimulus increases salivary flow stronger than the taste-neutral water control. It is hypothesized that the caloric load and concentration of a compound do not have a major impact on salivary flow, but the perception of sweet taste is the main driving force, independent of the type of sweetener. Bonnans and Noble (9) concluded in their study, with regard to salivary flow and the perception of sweet and sour, that the salivary flow response is not only based on the concentration of the stimulus but is also influenced by individual cognitive taste perception (9), which includes processed perception in the central nervous system. Because the stimulation was the strongest in the 1st min after stimulation (ΔT1), it reflects the fast adaptation previously described to a sweet stimulus (53, 54). A fast adaption process of salivary flow to food-derived stimuli was later also confirmed by the results in Criado et al. (55), demonstrating a stronger immediate effect of wine aroma on salivary flow than the long-lasting effect of the aroma. It has to be noted that, in this study, the sweet taste level of the test solutions was adjusted to be equivalent to 5% sucrose, which was previously demonstrated with trained subjects (28). Nevertheless, the test subjects rated the sweet taste of the test solutions to be different. One explanation could be the fact that the test subjects were sensorially naïve and were not specifically trained to differentiate the onset, maximum sweet taste, and lingering of a compound, whereas untrained panelists do present taste impressions from everyday society. Hence, it cannot be excluded that the test subjects confounded the well-known long-lasting lingering of NHDC and RebM (28) with enhanced maximum sweet taste, and trained panelists would have been able to distinguish this. It should be noted that RebM and NHDC showed longer lasting stimulation of salivary flow than sucralose, which is reflected by the increase in the flow rate in the 2nd min of stimulation (T2). This also argues for an effect of perceived sweet taste as NHDC and RebM are known for their long-lasting sweet taste, as described above. A reason could be that those compounds stick longer to the chemosensory surface, but this remains speculative. Further studies with trained panelists are needed to focus on the interaction of lingering and a long-lasting salivary flow, including a complete time-intensity profile and concentration dependence of the test compounds.

The second aim of our study was to explore a possible change of the salivary complex viscosity η after stimulation with sweet-tasting compounds. In general, saliva with low elasticity can lead to a moister mouthfeel, as demonstrated after the consumption of plain water (4). However, knowledge on the interactions of salivary rheology, sweet taste perception, and its mouthfeel remains scarce. Schipper et al. (56) summarized a wide range of studies investigating whole saliva viscosities: apparent viscosity η_a_ can range from 1.1 up to 10 mPas. The variation of values is based on the different methods applied (types of rheometer, shear rate, and temperature), collection and handling of saliva, circadian rhythm, type of glands, and individual variation (56). Therefore, especially the raw data can variate due to different study protocols and a comparison with raw data of other studies is difficult. However, the crossover design of our study allows us to compare the responses of the test persons to different stimuli. In contrast to our hypothesis, no differences between unstimulated and stimulated saliva or differences between treatments were found. As there were no differences in viscosity markers for unstimulated saliva on each taste day, we assume differences after stimulation with the test compounds—if any—to be below the limit of detection. The repeated measures ANCOVA excluded the influence of basal salivary pH and age of the test subjects. The statistical model revealed that, depending on the test compound, the complex viscosity is influenced by the interaction of elasticity and sweet taste threshold. Moreover, Muc5B had an impact on complex viscosity independent of the test compounds. Thus, the viscoelastic properties might affect the determination of the sweet taste threshold as well. After stimulation with sucrose, a higher storage modulus (*G*′), representing the elastic component of the saliva sample, was positively correlated with a higher sweet taste threshold. We hypothesize that this may be due to impaired transport of tastants to the taste pores as it has been shown for lower mixing efficiency in more elastic saliva (57). In this context, Ferry et al. (58) also showed that lowering the mixing efficiency by salivary amylase-released polysaccharides reduced the perceived saltiness. Those results support the importance of the interaction of the tastants with components in saliva (58). The protein precipitating properties of the sweeteners, especially polyphenolic structures like NHDC, could also contribute to the mouthfeel of sweeteners by following a similar mechanism as suggested for astringent sensations. Polyhydroxyphenols like tannins are proposed to aggregate the salivary pellicle, leading to the dissociation of the two subunits of the transmembrane protein MUC1, which causes pull out of the pellicle and neurotransmitter release (22). Karl et al. (28) have shown that NHDC and RebM show low but detectable astringent properties at the concentrations applied here. In addition, a potential direct interaction, especially of more complex sweeteners like NHDC and RebM, with salivary proteins present in mucosal pellicles, such as proline-rich proteins (PRPs), Muc5B, amylase, and cystatin, needs to be investigated in future studies. Muc5B was previously described to determine the viscosity of saliva (17, 59, 60) and this relation of Muc5B to η was confirmed here with a repeated measures ANCOVA, independent of the test compounds. Furthermore, the rheology of saliva depends on many inter- and intra-individual factors such as gender and hormonal balance (61), health status (62), and age (60). The rheological properties of food and saliva are constantly changing during the dynamic process of oral perception. Although we standardized sample collection and measurements and used a crossover design for the study, this dynamic process may be difficult to capture *in vivo* (5).

Another possible player in the taste perception and mouthfeel of sweet compounds and associated salivary parameters is the oral microbiome (25, 26). Especially, for the salivary pH, the role of oral microbiota is crucial because there are associations between sugar intake and oral microbiota ecology, and a variable microbiota response to sugar (25). The composition and diversity of oral microbiota shown here are typical of healthy human tongue samples (46–48). In line with previous studies of a healthy mouth environment (63), the composition of microbial communities was observed to be quite stable over time within an individual. However, we did not find an association between the oral microbiota and the analyzed salivary parameters. The impact of the oral microbiome is also discussed discordantly in the literature. On the one hand, Cattaneo et al. (64) associated one taxon with a negative correlation to the sweet taste threshold. A reason could be that less-sensitive individuals more frequently consume sweets and desserts. On the other hand, Feng et al. (65) did not find a correlation between sweet taste sensitivity and bacterial count in saliva and tongue salivary film, which corresponds to the findings of the present study with the same number of test subjects. This aspect needs further investigations with larger study populations to clarify the role of microbiota in sweet taste perception.

In this study, there are limitations related to sensory tasting and mouthfeel. First, we focused on the pure sweet taste impression of the test compounds and did not consider secondary tastes and temporal attributes, which led to an increased rating of sweet taste intensity for NHDC and RebM by sensorially naïve test subjects. In addition, future studies with trained panelists are needed to determine the impact of structure vs. sweet taste. The second limitation was a narrow range of characteristics such as BMI, basal salivary flow rate, age, and the limited number of test subjects. Furthermore, a moderate correlation between individually perceived sweet taste and the salivary flow rate in the 1st min (*r* = 0.3, *p* < 0.01) may be due to differences in water and sweet compounds. Thus, the association between the perception of sweet taste and flow rate, as well as possible associations of taste impression with the oral microbiome, needs to be verified in a larger study population. In addition, the pH effect of the tap water used in our study as a solvent for the tested sweet-tasting compounds remains unclear and needs to be specifically tested in future studies. Contrarily, the strengths of this study were that we included a wide variety of different influencing factors to ensure a broad overview on salivary characteristics and associated mouthfeel and, for the first time, included complex interactions between the different factors in the statistical analysis.

## Conclusion

The results presented in this study demonstrate that individual sweet taste perception after oral stimulation with sucrose, sucralose, NHDC, and RebM is associated with salivary flow, which indicates an impact of predominantly cognitive sweet taste impression on the salivary flow rate without measurable changes in the rheological properties of saliva. Nonetheless, the complex viscosity of saliva was influenced by Muc5B, as well as by an interaction of the test compounds with elasticity and sweet taste threshold. The results indicate that salivary flow and saliva viscoelasticity may contribute to the overall taste perception and mouthfeel and may affect the sensory profile of sweet-tasting compounds. This study provides a basis for further studies to understand the complex interaction of saliva and the sensory properties of sweet-tasting compounds.

## Data Availability Statement

The amplicon sequencing datasets presented in this study can be found in online repositories. The names of the repository/repositories and accession number(s) can be found below: https://www.ncbi.nlm.nih.gov/sra, BioProject ID PRJNA726851.

## Ethics Statement

The studies involving human participants were reviewed and approved by Ethics Committee of the University of Vienna (reference no. 00421; 2019). The patients/participants provided their written informed consent to participate in this study.

## Author Contributions

CK: conceptualization, data curation, formal analysis, investigation, methodology, software, validation, visualization, and writing—original draft, reviewing, and editing. AV: data curation, formal analysis, investigation, and writing—reviewing and editing. PP: conceptualization, formal analysis, investigation, methodology, resources, software, validation, visualization, and writing—original draft, reviewing, and editing. BH: data curation, formal analysis, software, validation, and writing—reviewing and editing. GS: methodology, resources, software, and writing—reviewing and editing. JL: conceptualization, funding acquisition, resources, supervision, and writing—reviewing and editing. DB: conceptualization, methodology, resources, and writing—reviewing and editing. JH: conceptualization and writing—reviewing and editing. MW: conceptualization, methodology, and writing—reviewing and editing. JK: resources and writing—review and editing. VS: conceptualization, resources, and writing—reviewing and editing. BL: conceptualization, funding acquisition, investigation, methodology, project administration, resources, supervision, validation, and writing—original draft, reviewing, and editing. All authors contributed to the article and approved the submitted version.

## Conflict of Interest

JL and JH were employed by Symrise AG, Holzminden, Germany. MW were employed by Symrise Distribution GmbH, Vienna, Austria. The remaining authors declare that the research was conducted in the absence of any commercial or financial relationships that could be construed as a potential conflict of interest.

## Publisher’s Note

All claims expressed in this article are solely those of the authors and do not necessarily represent those of their affiliated organizations, or those of the publisher, the editors and the reviewers. Any product that may be evaluated in this article, or claim that may be made by its manufacturer, is not guaranteed or endorsed by the publisher.

## Footnotes

1
https://www.hmpdacc.org/hmp/doc/HMP_MOP_Version12_0_072910.pdf

2
github.com/jfjlaros/demultiplex

3
https://cran.r-project.org/web/packages/vegan/vegan.pdf
