# Supplementary material for: Individual Sweet Taste Perception Influences Salivary Characteristics After Orosensory Stimulation With Sucrose and Noncaloric Sweeteners
Source: Front Nutr. 2022 May 25;9:831726. doi: 10.3389/fnut.2022.831726 (PMC9174746; doi:10.3389/fnut.2022.831726)
Supplement: Supplementary file 1 [file Data_Sheet_1.docx]

Supplementary Material


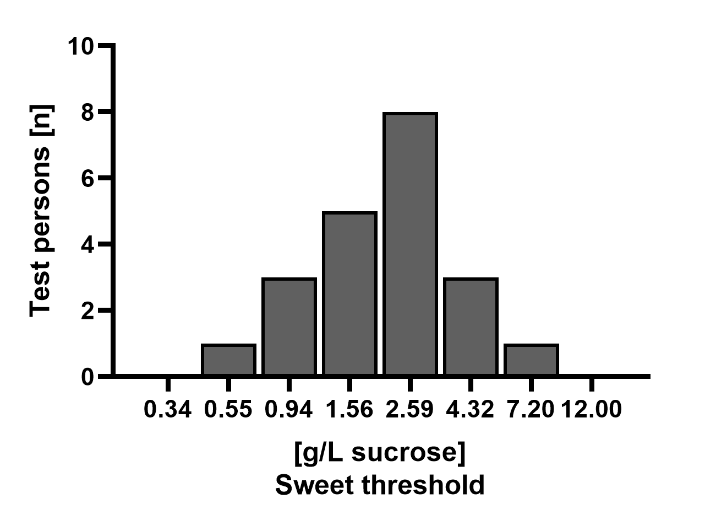


**Supplementary Figure S1:** Distribution of sweet sensitivity threshold [g/L sucrose] of test persons (n=21).

**Method establishment for rheological measurement of saliva**

**Supplementary Table S1:** Data of an amplitude sweep of fresh unstimulated saliva test sample at 36°C, with a 20 mm plate-plate probe, gap 0.5. The light green background highlights the linear region and dark green the selected shear strain (%).

| Complex shear strain (%) | Complex shear stress (Pa) | Shear modulus (complex comp.) (Pa) | Shear modulus (elastic comp.) (Pa) | Shear modulus (viscous comp.) (Pa) | Shear viscosity (complex comp.) (Pa s) | Phase angle (°) |
| --- | --- | --- | --- | --- | --- | --- |
| 0.101 | 0.001 | 0.862 | 0.592 | 0.626 | 0.274 | 46.560 |
| 0.215 | 0.002 | 0.781 | 0.623 | 0.470 | 0.249 | 37.020 |
| 0.464 | 0.004 | 0.834 | 0.605 | 0.575 | 0.266 | 43.540 |
| 1.000 | 0.008 | 0.834 | 0.648 | 0.526 | 0.266 | 39.090 |
| 2.156 | 0.016 | 0.753 | 0.515 | 0.550 | 0.240 | 46.890 |
| 4.643 | 0.027 | 0.588 | 0.315 | 0.496 | 0.187 | 57.570 |
| 9.999 | 0.040 | 0.398 | 0.094 | 0.387 | 0.127 | 76.420 |
| 21.544 | 0.057 | 0.263 | 0.060 | 0.256 | 0.084 | 90.000 |
| 46.417 | 0.095 | 0.205 | 0.137 | 0.153 | 0.065 | 90.000 |
| 99.995 | 0.196 | 0.196 | 0.174 | 0.090 | 0.062 | 90.000 |

**Results of ANCOVA (1)**

**Supplementary Table S2:** Results of the repeated measures ANCOVA for salivary flow (∆∆ T1) for test compounds with covariates: basal values (T0) for pH, α‑amylase activity, cystatin S (CysS) content as well as the sweet threshold, body height, BMI and age of test subjects. Impact of covariances to intrasubject factor and intermediate subject effects are shown with p values. Mauchly test for sphericity passed (p> 0.05).

| Source | **Intrasubject factor**  **(p value)** | Source | **Intermediate subject effects (p value)** |
| --- | --- | --- | --- |
| TestCompound | 0.508 | Constant term | 0.244 |
| TestCompound * Amylase.T0 | 0.600 | Amylase.T0 | 0.160 |
| TestCompound * CysS.T0 | 0.377 | CysS.T0 | 0.659 |
| TestCompound * pH.T0 | 0.828 | pH.T0 | 0.197 |
| TestCompound * Threshold | 0.484 | Threshold | 0.109 |
| TestCompound * BodyHeight | 0.866 | BodyHeight | 0.081 |
| TestCompound * BMI | 0.250 | BMI | 0.287 |
| TestCompound * Age | 0.769 | Age | 0.474 |
| TestCompound * Threshold * BMI | 0.396 | Threshold * BMI | 0.169 |
| TestCompound * Amylase.T0 * Threshold | 0.737 | Amylase.T0 * Threshold | 0.096 |
| TestCompound * CysS.T0 * Threshold | 0.140 | CysS.T0 * Threshold | 0.774 |
| TestCompound * Threshold * Age | 0.728 | Threshold * Age | 0.492 |

**Viscoelasticity: G´ and G´´ raw data**

**Supplementary** **Table S3**: Data of G´ storage modulus and G´´ loss modulus. One value displays the mean ± SEM of 21 saliva samples, measured with 2-3 repetitions and computed from the mean of a linear frequency range of 0.5 - 0.2.

| **Test solution** | **Time point** | **G´ storage modulus** | | | | **G´´ loss modulus** | | | |
| --- | --- | --- | --- | --- | --- | --- | --- | --- | --- |
|  |  | MW | ± | SEM | MW | | ± | SEM |  |
| Water | T0 | 7.58 | ± | 1.45 | 9.75 | | ± | 1.09 |  |
|  | T1 | 6.48 | ± | 0.78 | 9.20 | | ± | 0.96 |  |
|  | T2 | 9.67 | ± | 2.30 | 10.95 | | ± | 1.47 |  |
| NHDC | T0 | 5.94 | ± | 0.85 | 8.85 | | ± | 0.80 |  |
|  | T1 | 5.52 | ± | 0.67 | 8.74 | | ± | 0.91 |  |
|  | T2 | 6.69 | ± | 0.93 | 9.05 | | ± | 0.84 |  |
| RebM | T0 | 9.34 | ± | 1.19 | 10.92 | | ± | 0.39 |  |
|  | T1 | 9.76 | ± | 1.57 | 11.08 | | ± | 0.51 |  |
|  | T2 | 11.06 | ± | 1.97 | 10.95 | | ± | 0.65 |  |
| Sucralose | T0 | 11.19 | ± | 2.89 | 10.85 | | ± | 1.39 |  |
|  | T1 | 10.35 | ± | 2.72 | 10.03 | | ± | 1.38 |  |
|  | T2 | 10.51 | ± | 2.42 | 9.91 | | ± | 1.22 |  |
| Sucrose | T0 | 6.38 | ± | 1.03 | 8.39 | | ± | 0.90 |  |
|  | T1 | 8.32 | ± | 1.63 | 8.70 | | ± | 0.83 |  |
|  | T2 | 12.08 | ± | 3.13 | 10.16 | | ± | 1.15 |  |

**Supplementary** **Figure S2:** Storage modulus (G´), loss modulus (G´´) and phase angle (δ) versus frequency of saliva samples: example of the original data. Samples of one test person before (T1) and after (T1, T2) stimulation with sucrose, each tested with two separate measurements.

**Results of ANCOVA (2)**

**Supplementary Table S4:** Results of repeated measures ANCOVA for complex viscosity η (∆∆ T1) for test compounds with covariates: basal values (T0) of MUC5B, pH, α-amylase, storage modulus (G´) and phase angle (δ) of saliva as well as sweet threshold and age of test subjects. Impact of covariances to intrasubject factor and intermediate subject effects are shown with p values. Mauchly test for sphericity passed (p> 0.05).

| Source | **Intrasubject factor**  **(p value)** | Source | **Intermediate subject effects (p value)** |
| --- | --- | --- | --- |
| TestCompound | 0.710 | Constant term | 0.240 |
| TestCompound * MUC5B.T0 | 0.474 | MUC5B.T0 | 0.033 |
| TestCompound * G´.T0 | 0.778 | G´.T0 | 0.204 |
| TestCompound * δ.T0 | 0.656 | δ.T0 | 0.054 |
| TestCompound * pH.T0 | 0.701 | pH.T0 | 0.200 |
| TestCompound * Amylase.T0 | 0.786 | Amylase.T0 | 0.073 |
| TestCompound * Threshold | 0.693 | Threshold | 0.231 |
| TestCompound * Amylase.T0 * Threshold | 0.569 | Amylase.T0 * Threshold | 0.091 |
| TestCompound * pH.T0 * Threshold | 0.822 | pH.T0 * Threshold | 0.220 |
| TestCompound * δ.T0 * Threshold | 0.098 | δ.T0 * Threshold | 0.154 |
| TestCompound * G´.T0 * Threshold | 0.047 | G´.T0 * Threshold | 0.410 |
| TestCompound * MUC5B.T0 * Threshold | 0.312 | MUC5B.T0 * Threshold | 0.076 |
| TestCompound * Threshold * Age | 0.196 | Threshold * Age | 0.602 |

**Oral Microbiome**


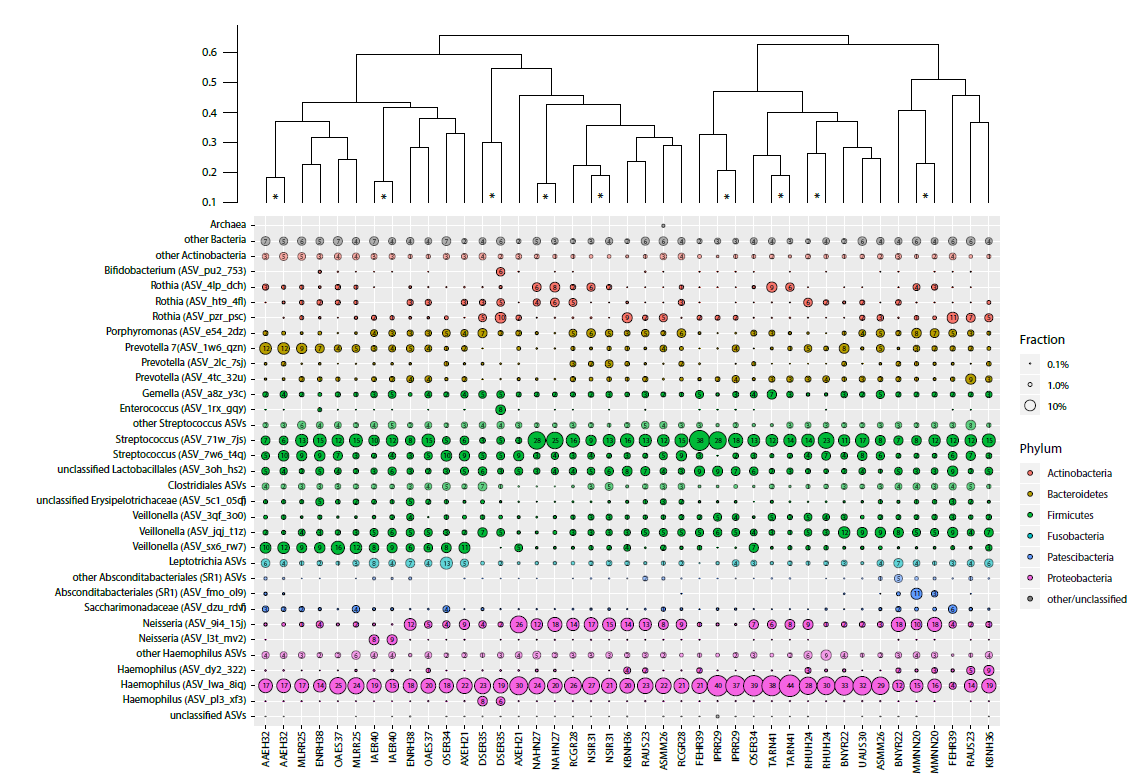


**Supplementary** **Figure S3:** Taxonomy overview bubbles sorted by clustering. Numbers in bubbles are percentages when relative abundance exceeded 1%. Faded bubbles are the sum of multiple ASVs at higher taxonomic ranks. Fractions shown for higher taxonomic ranks are exclusive of the fractions for separately shown lower taxonomic ranks. Parameters for collapsing taxa: relative abundance <5%, read count < 9. Solid bubbles are single ASVs.
